# Potentially Important Therapeutic Interactions between Antibiotics, and a Specially Engineered Emulsion Drug Vehicle Containing Krill-Oil-Based Phospholipids and Omega-3 Fatty Acids

**DOI:** 10.3390/antibiotics7010022

**Published:** 2018-03-10

**Authors:** David F. Driscoll

**Affiliations:** Stable Solutions LLC, Goleta, CA 93117, USA; d.driscoll@stablesolns.com

**Keywords:** β-lactam antibiotics, β-lactamase inhibitors, krill oil phospholipids, omega-3 fatty acid triglycerides, antimicrobial resistance

## Abstract

The incidence of antimicrobial resistance (AMR) worldwide is increasing as the pipeline for the development of new chemotherapeutic entities is decreasing. Clearly, overexposure to antibiotics, including excessive dosing, is a key factor that fuels AMR. In fact, most of the new antibacterial agents under development are derivatives of existing classes of antibiotics. Novel approaches involving unique antimicrobial combinations, targets, and/or delivery systems are under intense investigation. An innovative combination of active pharmaceutical ingredients (APIs) consisting of antimicrobial drug(s), krill-oil-based phospholipids, and omega-3 fatty acid triglycerides, that may extend the therapeutic viability of currently effective antibiotics, at least until new chemical entities are introduced, is described.

## 1. Introduction

Antimicrobial resistance (AMR) is a worldwide concern. The United Nations, the WHO, and major pharmaceutical companies are committed to reducing AMR, with the latter providing a roadmap to achieve this goal by the year 2020 [1]. The roadmap includes four key commitments: (1) reduce the environmental impact of the production of antibiotics; (2) use antibiotics only in patients who need them; (3) improve access to existing and future antibiotics (including diagnostics and vaccines); (4) promote open collaborations between industry and public researchers. The fourth commitment broadly describes product development and the facilitation of the exchange of data on old antibiotics in efforts to fill specific gaps in the global pipeline.

Moreover, there has not been a successful pharmaceutical development of a newly discovered class of antibiotics in over 30 years. Therefore, all newer antibiotics that have reached the market since then have mostly been minor physicochemical modifications of existing drugs [2]. Consequently, if AMR exists to a certain class of antibiotics, it will eventually affect the modified congener that exhibits a similar structure–activity relationship. Therefore, alternative pharmaceutical strategies are being devised to optimize the delivery of particular antimicrobial agents or related APIs, or a combination of APIs. A more integrated approach that includes other pharmaceutical factors seems to be a reasonable additional consideration during drug development to reduce the incidence of AMR. Such factors include (a) precise dosing, (b) combination API therapy, (c) novel drug delivery systems, (d) shortened treatment intervals, and (e) restricted usage. The focus of this review will center on combination API therapy and novel drug delivery systems. In particular, an intravenous oil-in-water delivery system consisting of a combination of APIs will be described in the setting of bloodstream infections in the intensive care unit (ICU). Importantly, a major factor affecting the morbidity and mortality rates of septic ICU patients is the intensity of the accompanying systemic inflammatory response syndrome (SIRS).

A roadmap has recently (May 2016) been laid out by a multidisciplinary group of leading industry and academic experts “to identify the key scientific roadblocks to antibiotic discovery,” supported by, and contained in, the Pew Charitable Trusts Report [2]. As of July 2016, of the 43 small molecule antibiotics under development, only 6 in the “pipeline” represent novel classes, and only one of which exhibits activity against Gram-negative bacteria [3]. A major emphasis in antibiotic research and development remains focused on the combination therapy of β-lactam antibiotics coupled with β-lactamase inhibitors (BLI), i.e., the application of two APIs. For example, the more recent antibiotic approvals for Gram-negative microorganisms, i.e., in 2014, US: ZERBAXA™ (ceftolozane–tazobactam) and, in 2015, US: AVACAZ™ (ceftazidime–avibactam), are BLI combination products consisting of a cephalosporin antibiotic (e.g., ceftazidime) and a BLI (e.g., avibactam). Although they provide better coverage against Gram-negative microorganisms vs. monotherapy, resistance has been observed [4]. Similarly, the first such combination therapy with penicillins, which are structurally related to cephalosporins and within the class of β-lactam antibiotics, was previously introduced 15 or more years ago, i.e., in 1997, US: UNASYN™ (ampicillin–sulbactam) and, in 2003, AUGMENTIN™ (amoxicillin–clavulanic acid), but the subsequent resistance to these therapies eventually led to the development of ZERBAXA™ and AVACAZ™.

Clearly, in the absence of a scientific breakthrough of new chemical classes of antimicrobial agents, alternative pharmaceutical strategies seem necessary. Over the last 30 years or so, pharmacokinetic (PK) techniques have been applied to support the optimal doses of antibiotics. Pharmacokinetics focuses on the time course of drug concentrations primarily in plasma, whereas pharmacodynamics (PD) relates the concentration of a drug in plasma to the observed pharmacological effect. Presently, combining PK/PD into a single statistical model is now routinely performed to assess the time course of the drug and the influence on its desired pharmacological effects, and this approach may be a more optimal way to dose BLI combinations [5]. Other proposed strategies in the search for viable antibiotic therapies for bloodstream infections include novel targeting agents and drug delivery systems [3]. Recent advancements in the analytical techniques applied to nanotechnology that allow more precise control of particle or droplet size distributions [6] have greatly added to the development of injectable dosage forms.

## 2. Bloodstream Infections in the ICU: SIRS, Sepsis, and Endotoxin—A Lethal Combination

Gram-negative sepsis is one of the most lethal bloodstreams infection in the ICU, and common pathogens include *Escherichia coli*, *Klebsiella pneumoniae*, and *Pseudomonas aeruginosa*. It is particularly lethal because the endotoxin or lipopolysaccharide produced accentuates the severity of the infection. Endotoxin, a component of the bacterial cell wall, is recognized by Toll-like receptors (TLRs) by the host, which binds this component and triggers activation of the nuclear factor kappa or the NFκB pathway, which in turn stimulates the synthesis and release of pro-inflammatory cytokines (e.g., tumor necrosis factor alpha (TNFα), interleukin-1 beta (IL-1β), and interleukin-6 (IL-6)) and stimulates other aspects of the immune system that increase the intensity of the metabolic response to infection. Thus, a vicious cycle ensues involving three main drivers that underlie the high mortality rate associated with Gram-negative sepsis: (1) SIRS, (2) sepsis, and (3) endotoxinemia. Consequently, using Gram-negative sepsis as a treatment model, a brief summary of these three variables (and later a sample intravenous combination-therapy regimen) is described.

### 2.1. Systemic Inflammatory Response Syndrome (SIRS)

In the intensive care unit (ICU) setting, all patients exhibit an acute inflammatory phase arising from high metabolic stresses affecting the function of vital organs (e.g., lungs, kidneys, heart, liver, and brain). If left unabated, the resulting multi-organ failure may produce a pronounced systemic inflammatory response syndrome (SIRS). As a key component of the metabolic response to injury, the increased endogenous production of eicosanoids (prostaglandins, thromboxanes, leukotrienes, and their downstream bioactive mediators, resolvins and protectins) is a vital part of the host response to SIRS. This heightened metabolic activity elicits an immune response that includes the production of other pro-inflammatory mediators (cytokines, chemokines, adhesion molecules, and reactive oxygen species) [7]. Hence, as inflammation increases, changes in selected (acute phase) proteins in plasma are also observed. Most notably, there is a reduction in the serum albumin level (resulting from extravascular pooling and heightened catabolism), as well as elevation of the C-reactive protein (CRP). As CRP is an acute phase protein, the magnitude of the increase in CRP is a reflection of the extent of metabolic and infectious-related stress, as well as tissue injury. When SIRS is accompanied by sepsis, the mortality rate progressively increases. There are numerous non-infectious causes as well, such as burn injury to >30% of body surface area, hemorrhagic shock, myocardial infarction, pancreatitis, and upper gastrointestinal bleeding. Similarly, there are numerous infectious causes of SIRS in the ICU, which include bacteremia, candidiasis, infectious endocarditis, pneumonia, and urinary tract infections. The clinical manifestations of SIRS in critically ill patients include the following four standard benchmarks (where diagnosis requires at least two of four to be present) in the diagnosis of this life-threatening illness [8]:

(1) a temperature of >38 °C or <36 °C;
(2) a heart rate of >90/minute;
(3) a respiratory rate of >20/minute (or PaCO_2_ < 32 mmHg);
(4) a white blood cell count of >12,000/mm^3^ or <4000/mm^3^ (or 10% immature bands).

Once SIRS is diagnosed, additional pathological insults (i.e., co-morbid diseases) can lead to a deteriorating clinical state (i.e., known as the SIRS “cascade”) that progressively increases the risk of mortality (%) that may start from a non-infectious source of SIRS (7%), progressing to an infectious SIRS insult: sepsis (16%) → severe sepsis (20%) → septic shock (46%) [9]. Current therapeutic approaches to SIRS include supportive medical therapies such as coronary vasodilation therapy, anti-ulcer regimens, appropriate antimicrobial therapy, blood product replacement, and even, for example, the strategic placement of surgical drains. Blocking the enhanced production of eicosanoids derived from omega-6 fatty acids by inhibiting the key enzymes that produce them (i.e., cyclooxygenases and/or lipoxygenases) is one way to reduce the intensity of SIRS. This has been achieved in critical care settings with injectable dosage forms of non-steroidal anti-inflammatory drugs (NSAIDs), such as ketorolac, which inhibits prostaglandin synthesis (i.e., selectively blocks cyclooxygenase-1 enzyme). However, in so doing, the drug increases the risk of damage to the gastrointestinal tract, the cardiovascular system, and the kidneys, and this is specifically detailed in the FDA’s black-box warning (i.e., the strongest warning by the FDA for a drug with serious or fatal adverse reactions) that accompanies the package insert for ketorolac injection. Therefore, the NSAID therapy is currently viewed as potentially harmful in critically ill patients, and is not routinely applied in this setting. Additionally, metabolic therapies for SIRS include providing lipid substrates (essential fatty acids) that will modify the type of endogenous mediators that are produced, which fuel the inflammatory response—in particular, the production of prostaglandins (PGs), thromboxanes (TXAs), and leukotrienes (LKTs), i.e., the eicosanoids. Modifying the type of eicosanoids produced (e.g., from pro-inflammatory 2-series PGs and 4-series LKTs from omega-6 fatty acids to anti-inflammatory 3-series PGs and 5-series LKTs from omega-3 fatty acids) has been achieved by acutely altering the lipid substrate provided during the acute phase response. In this regard, the principal pro-inflammatory omega-6 fatty acids of interest are arachidonic acid (ARA) and linoleic acid (LA), while the key anti-inflammatory omega-3 fatty acids of interest are eicosapentaenoic acid (EPA) and docosahexaenoic acid (DHA). Modulating the intensity of the inflammatory response by altering the type of eicosanoids produced via intravenous infusion of omega-3 fatty acids seems to be an attractive alternative. For example, in a randomized controlled trial, 28 patients undergoing elective cardiopulmonary bypass surgery who received three perioperative intravenous lipid infusions containing omega-3 fatty acids had decreased biological and clinical signs of inflammation [10]. The acute provision of omega-3 fatty acids will be effectively incorporated into plasma cell membranes, and, as the preferred substrate over omega-6 fatty acids for the cyclooxygenase and lipoxygenase enzymes, such acids will ultimately produce anti-inflammatory eicosanoids. Importantly, providing omega-3 fatty acids intravenously greatly improves their bioavailability, i.e., changes in membrane composition (within 60 min of infusion [11]), over oral supplementation (requiring 6–8 weeks [12]). Thus, the anti-inflammatory effects can be achieved quickly—an absolute requirement in the clinical management of significant SIRS in the critical care setting.

### 2.2. Sepsis and Endotoxin

Blood infection or sepsis is a major cause of death in critically ill patients. Of those who survive, according to the Third International Consensus Definitions for Sepsis and Septic Shock (Sepsis-3) on 23 February 2016, they have “long-term physical, psychological, and cognitive disability with significant health care and social implications. […] Sepsis is defined as the life-threatening organ dysfunction caused by a dysregulated host response to infection. […] In lay terms, sepsis is a life-threatening condition that arises when the body’s response to an infection injures its own tissues and organs” [13]. In the ICU, sepsis is a major form of metabolic stress, so it is associated with a heightened level of the systemic inflammatory response in ICU settings and often involves organ dysfunction.

In May 2016, a report from the Pew Charitable Trusts entitled “A Scientific Roadmap for Antibiotic Discovery” [2] acknowledges the fact that, for more than 30 years, there has been a “void” in the discovery of new classes of antibiotics. Not surprisingly, coinciding with this drought in pharmaceutical development (i.e., “no registered classes of antibiotics since 1984”), there has been a dramatic rise in antibiotic resistance (ABR), as noted in the declaration by the WHO that characterized this timespan as a “post-antibiotic era.” A major priority is “understanding and overcoming barriers for drugs targeting Gram-negative bacteria,” which is a leading cause of septic morbidity and mortality in ICU patients. In Gram-negative sepsis, endotoxins or lipopolysaccharides are the prime cause of increased morbidity and mortality because they trigger the immune response that includes the pronounced secretion of inflammatory mediators This is because the intensity of the metabolic response to injury and/or infection is a reflection of the patient’s diet, and consumption of omega-6 rich soybean oil worldwide has been increasing. Consequently, if left unaddressed in the ICU patient, it can lead to septic shock with myocardial depression and renal impairment with mortality rates approaching 50% or higher. Although targeted API injectable therapies for bloodstream infections, such as those that antagonize or remove endotoxin from the bloodstream, seems to be a reasonable approach, there have been significant setbacks. For example, in 2009, an anti-endotoxin therapy was tried with a 10% phospholipid (PL-10) injectable emulsion (92.5 mg/mL of phosphatidyl choline, 7.75 mg/mL of sodium cholate, and 7.5 mg/mL of soybean oil) in two doses, compared to a placebo group in patients with confirmed or suspected Gram-negative severe sepsis [14]. The hypothesis was that the phospholipids would bind and neutralize endotoxin and lead to improved clinical outcomes. A total of 235 centers worldwide participated in the study, and the injectable emulsion was added to a pre-existing antibiotic regimen. Although the results were positive in previous large animal models of endotoxinemia (including one infection model) as well as during endotoxin challenge in normal human volunteers, the high phospholipid dose arm of the study (1350 mg or 13.5 mL of PL-10/kg; *n* = 182) was stopped on the recommendation of an Independent Safety Monitoring Committee due to an increase in life-threatening, serious adverse events detected in the fourth interim analysis. However, patient entry was completed in the lower dose arm (850 mg (or 8.5 mL of PL-10/kg; *n* = 598) and the placebo (*n* = 599) groups, but there were no differences in mortality, nor in the onset of new organ failure. More recently, there is another anti-endotoxin proposal that is being investigated, which involves coupling an antimicrobial drug effective against Gram-negative organisms with an extracorporeal blood purification technique [15]. Here, the antimicrobial drug polymyxin B is embedded in a polystyrene fiber and attached to a hemoperfusion device [16], but now, it appears to be no more effective than standard therapy [17].. In another novel approach, designed to mitigate the risk of mortality (based on an APACHE II score of ≥25) in adult ICU patients with severe sepsis, the intravenous administration of a recombinant form of human activated protein C (XIGRIS™) for 96 h was investigated and was reported to exert an anti-inflammatory effect by decreasing the leukocyte response to inflammatory cytokines (e.g., IL-6), resulting in reduced mortality [18]. However, in a follow-up study [19], the results could not be confirmed. Consequently, although there were no safety issues associated with the APIs in this study, the lack of clinical benefits prompted the manufacturer to withdraw the drug from the market on 25 October 2011.

## 3. New Therapeutic Combinations: Antibiotics + BLIs + Phospholipids + Omega-3 Fatty Acids

In addition to continuing to refine and, in some cases, develop more specific PK/PD models for a particular drug and/or dosage form, a novel drug delivery system has been developed, containing, for example, three or more APIs, as described below. What makes this dosage form unique is the use of ingredients that would normally be considered excipients (i.e., providing pharmaceutical stability and compatibility) but are used to exploit inherent pharmacological actions that exhibit API-related effects. The goal of course is to use them along with the primary API (antibiotic) in a synergistic manner that may reduce its dose (exposure), as well as favorably influence the clinical response to sepsis. That is, the excipient(s) to be included have an important dual function—a pharmaceutical role and a therapeutic role as an API. The pharmaceutical and pharmacological roles of the proposed intravenous formulation for Gram-negative sepsis is described below in Table 1.

Historically, the primary use of lipid injectable emulsions in the clinical setting have been for nutritional purposes, i.e., to deliver a dense source of fat calories and essential fatty acids. Subsequently, they were used as drug delivery vehicles for poorly water-soluble drugs such as propofol injectable emulsion. More recently, focus has been placed on the fatty acid composition of the oil phase with respect to the omega-3 and omega-6 fatty acid contents, and in particular, the effects on the systemic inflammatory response. Increasing the concentration of omega-3 fatty acids delivered during the acute phase of critical illness has an anti-inflammatory effect that may improve clinical outcomes [7]. Currently, a novel approach is to include polyfunctional components that have both pharmaceutical and pharmacological functions.

### 3.1. Polyfunctional Ingredients

#### 3.1.1. Krill Oil Phospholipids

All phospholipids have the same unique character of possessing two hydrophobic nonpolar tail groups (sn-1 and sn-2 of the triglyceride structure) and a hydrophilic polar head group (sn-3 of the triacylglycerol structure) that imparts surface-active properties, i.e., a pharmaceutically effective emulsifier for the homogeneous dispersion of oil droplets in oil-in-water (o/w) injectable emulsions. This molecular arrangement allows the hydrophilic head groups and hydrophobic tail groups to align along the oil–water interface of the submicron droplets in a tight packing arrangement in order to electromechanically stabilize the emulsion. Krill oil phospholipids (KO-PLs) are unique compounds in that they are a marine-based PL and, as such, uniquely contain anti-inflammatory 20+ carbon, polyunsaturated omega-3 fatty acids EPA (20:5n3) and DHA (22:6n3). In contrast, the conventional PL from egg yolk phosphatides that come from hens that are fed vegetable grains high in pro-inflammatory 18-carbon omega-6 fatty acids and saturated fatty acids. In terms of the egg phospholipid fatty acid profile, it typically contains fatty acids—in decreasing order, palmitic (16:0), oleic (18:1n9), stearic (18:0), and linoleic (18:2n6) acids—that are attached to the available sn-1 and sn-2 positions, whereas 70–95% of the omega-3 fatty acids EPA (20:5n3) and DHA (22:6n3) are attached to the phospholipids in krill oil. This difference may have significant and unique implications for pharmaceutical actions of KO-PLs as emulsifying and/or solubilizing agents. For example, we know in living organisms that both EPA and DHA are not only the longest-chain fatty acids found in biological membranes, but they are also the most unsaturated (5 and 6 double bonds, respectively). They are highly flexible (having elastic compressibility) and can interconvert between multiple torsional states that can alter membrane order and fluidity. In pharmaceutical emulsions, these characteristics can uniquely alter the molecular packing arrangement that influences not only the shape of micelles, but also their surfactant properties. The “packing parameter P” is certainly influenced by the long hydrophobic tail, and this may be especially important with respect to the unique ability of omega-3 fatty acids to enhance their surfactant properties over shorter chain fatty acids with lower numbers of carbons, and degrees of unsaturation, or with saturated long-chain fatty acids, typically found in conventional egg phospholipids. These differences may be especially important for highly water-insoluble drugs intended for intravenous administration. Pharmaceutically, KO-PLs have potentially useful surfactant and drug-solubilizing activities.

Although krill oil is not a high source of omega-3 fatty acids (up to 35% of the fatty acid profile) compared to enriched fish oil (≥45%), it nonetheless makes an important contribution to the omega-3 fatty acids mainly provided by highly enriched fish oil triglycerides. This is a key consideration when EPA + DHA are provided in pharmacological doses in the treatment of SIRS that accompanies sepsis. In addition, as phospholipids, KO-PLs are capable of binding endotoxin produced during Gram-negative sepsis. In cell culture studies, KO-PL emulsions have been shown to protect against endotoxin-induced pro-inflammatory activation of macrophages [20]. As with any drug dose, and as described earlier, injectable phospholipids in high concentrations can be dangerous. In the Dellinger et al. study [14], however, the concentration of PL-10 emulsion that was infused was nearly an order of magnitude higher (10%) than the PL concentrations that have been safely given in injectable nutritional and drug emulsions (1.2%). Such differences (~log dose), as observed in typical dose-finding studies of any drug, often reveal the toxicity of many compounds. Pharmacologically, in safe and effective doses, KO-PLs have demonstrated potentially useful activity against SIRS and endotoxinemia. The safe and effective clinical dose of krill oil phospholipids will require future study, but for emulsion stability, concentrations between 0.6 and 1.8% have been safely given since the introduction of nutritional emulsions in 1961.

#### 3.1.2. Fish Oil Triglycerides Enriched in Omega-3 Fatty Acids

According to the European Pharmacopoeia (EP), there are two pharmaceutical monographs for fish oil: EP Monograph 1352 entitled “Omega-3 Acid Triglycerides” and EP Monograph 1912 entitled “Fish Oil, Rich in Omega-3 Acids” [21]. EP 1912 is a natural fish oil that has been refined and made appropriate for human consumption. Of the total fatty acid profile, it contains about 30% (by weight) of the essential fatty acids EPA and DHA. Typically, this type of fish oil is contained in fish oil capsules used as oral supplements. In contrast, EP1352 is both refined and enriched (e.g., by molecular distillation) to increase the concentration of EPA and DHA to about 60% of the total fatty acid profile. This type of fish oil is often used in injectable emulsions. The oil phase of conventional lipid injectable emulsions plays a key role as a carrier for poorly water-soluble drugs. An internal or dispersed phase consisting of mainly very long-chain (20–22 carbons) triglycerides of fish oil may assist in the solubilization of practically water-insoluble drugs, where conventional long-chain (16–18 carbons) triglycerides are not as effective. Pharmaceutically, fish oil triglycerides that are highly enriched with omega-3 fatty acids may result in improved delivery of the drug and/or pharmaceutical stability compared with conventional oils (soybean oil, medium chain triglyceride (MCT) oil, olive oil, etc.) used in intravenous lipid emulsions. Consequently, enriched fish oil triglycerides are the major source of omega-3 fatty acids in addition to those present in KO-PLs. When dosing, the effort should be focused on the absolute intakes of EPA + DHA and not on the omega-6/omega-3 ratio. The optimal intravenous dose that is effective in the treatment of SIRS, in the presence of sepsis, has not been determined. Based on an assessment of the literature on omega-3 fatty acids used to treat various inflammatory diseases, such as cardiovascular disease [22], a threshold dose of about 2 g of EPA + DHA per day appears to be necessary. It is worth noting that it may take several weeks of oral supplementation before clinical benefits are seen [7]. In contrast, the bioavailability of intravenous omega-3 fatty acids is fast (within 60 min of a bolus dose [11]). Therefore, close attention must be paid to identifying the ideal pharmacological dose range, ensuring that it is both safe and effective upon intravenous infusion in critically ill patients with SIRS + sepsis. The daily dose of omega-3 fatty acids necessary to favorably alter the clinical course of SIRS during sepsis will require future study, but intravenous doses of fish oil in the range of 1–6 g/day of EPA+DHA have been safely provided in ICU patients [23,24]. Furthermore, in patients with renal failure, the dose alterations for omega-3 fatty acids will likely parallel the modifications made for antibiotics, but this too will require future study.

#### 3.1.3. Medium Chain Triglyceride Oil

**Figure 1:**
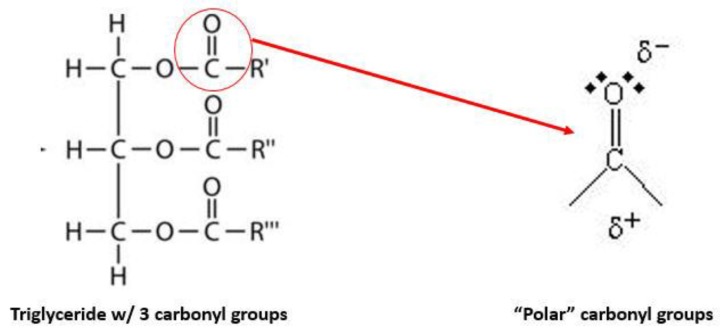
Triacylglycerol backbone and polar carbonyl groups.

MCTs consist of saturated fatty acids (no double bonds) containing between 6 and 12 carbon atoms. For use in lipid injectable emulsions, more than 95% of the fatty acids present are caprylic (8:0) and capric (10:0) acids (occupying the R’, R”, and R’” below). Although triglyceride molecules are highly hydrophobic and therefore referred to as “neutral triglycerides,” all of them possess three carbonyl groups that impart a slight polarity to the structure, as illustrated in Figure 1.

These carbonyl structures allow triglycerides (to varying degrees) to be incorporated into the phospholipid bilayers of the egg lecithin surfactant at the oil–water interface. The shorter the hydrocarbon chain is, compared to the longer hydrocarbon chains that will orient to the oil phase, the closer the fatty acid will be to the aqueous phase. In a fish oil–MCT oil mixed emulsion, the unique MCT orientation has a significant impact on the physiochemical properties of the emulsion. It has been shown that MCTs displace Long Chain Triglycerides (LCTs) from the oil–water interface, and this behavior influences the intensity of the chemical stress imposed upon the emulsifier, which reduces the interfacial tension between the internal lipid phase and the external aqueous phase. For example, the interfacial tension of the 18-carbon oleic acid is 15.6 dyne/cm vs. 8.22 dyne/cm for the 8-carbon caprylic acid. Consequently, the stress upon the emulsifier at this oil–water interface is lower when MCTs are present. In fact, the pharmaceutical stability of lipid injectable emulsions is superior when MCTs are included with LCTs in mixed-oil emulsions [25]. Clinically speaking, these unique surface-active effects allow for the greater interaction with water-soluble proteins, such as the enzyme lipoprotein lipase, resulting in faster hydrolysis and clearance of mixed-oil lipid droplets upon infusion into the bloodstream [26]. That is, the longer the hydrocarbon chain length of the fatty acids present, the slower the clearance of lipid emulsions from the bloodstream, which may increase the risks of adverse events, especially in an ICU setting [21]. Therefore, from a pharmacological perspective, the presence of MCTs with fish oil triglycerides in lipid injectable emulsions improves the bioavailability and safety of the infusion.

#### 3.1.4. Sodium Oleate

Of the five main free fatty acids present in the soybean oil-in-water injectable emulsion INTRALIPID™ (52% linoleic, 22% oleic, 13% palmitic, 8% linolenic, and 4% stearic), oleic acid is best known to exhibit surfactant properties. Whether or not this is related to its unique chemical structure (a single, *cis*-configured, double bond at the center of the hydrocarbon chain) is not known, but its increased presence in “aged” INTRALIPID emulsions improves its physical stability [27]. The fatty acids attached to the triglycerides in soybean oil-in-water emulsions will be hydrolyzed over time, and, because free fatty acids are potentially toxic upon intravenous administration (causing, for example, blood and liver abnormalities, pulmonary edema, ventilator defects, and kernicterus) [28], their formation must be limited during the shelf life of the product. Oleic acid’s corresponding fatty acid salt (sodium oleate), however, is a safe excipient that retains the co-emulsifying properties and has been used for many years in commercially available lipid injectable emulsion products. Pharmaceutically speaking, along with the primary emulsifier, i.e., the surface-active phospholipids in krill oil, such products are the main stabilizers of the intravenous emulsion and allow sodium oleate to be safely infused. Anything that compromises the surfactant properties will promote the coalescence of submicron lipid droplets into large-diameter, and potentially embolic, fat globules that can occlude the pulmonary microvasculature (e.g., capillaries). As a consequence of its surface-active properties, sodium oleate may also play a complementary or synergistic role with phospholipids in the neutralization of endotoxin, but this has not been proven at this time. If true, this excipient also plays a therapeutic role in the formulation.

#### 3.1.5. Alpha Tocopherol

From a pharmaceutical standpoint, alpha tocopherol or vitamin E is a critical stabilizing component routinely included in lipid injectable emulsions. This is particularly true if unsaturated fatty acids are present in the triglycerides that make up the oil phase of the emulsion. With the exception of MCT oil, all commonly used triglyceride oils contain unsaturated fatty acids. As the number of double bonds in the fatty acid increases, so too does the risk of oxidative degradation. For example, oleic acid is a monounsaturated fatty acid containing a single double bond; linoleic acid contains two double bonds; linolenic acid contains three; ARA has four, EPA has five, and DHA has six. Key measures in emulsions include the primary oxidation product (peroxide value) and the secondary oxidation product (anisidine), and these are routinely monitored over the course of the shelf life of the product. Importantly, given the highly polyunsaturated fatty acids present in fish oil used to treat SIRS, alpha tocopherol is a vital pharmaceutical excipient that preserves the quality and safety of the final injectable emulsion.

In addition, alpha tocopherol may have a systemic effect as an antioxidant. During severe critical illness and SIRS, oxidative stress is one of four hallmark events that need to be addressed (in addition to inflammation, ischemia, and immune dysfunction). Thus, the provision of alpha tocopherol may have a complementary therapeutic effect along with the omega-3 fatty acids in treating SIRS. However, this potential benefit, and the optimal dose needed to achieve both pharmaceutical and pharmacological benefits, has not yet been identified.

## 4. Conclusions

AMR is a clinically significant problem worldwide with lethal consequences, prompting numerous and diverse organizations in the search for therapeutic strategies in critically ill patients with bloodstream infections. Due to the lack of development of a new class of antibiotics in over 30 years, pharmaceutical methods may enhance pharmacological efficacy. For example, improvements in our understanding of PK/PD modeling, a novel dosage form design, and combination antimicrobial therapies may extend the activity against some of the most lethal microorganisms (e.g., Gram-negative bacteria) responsible for sepsis. In doing so, such pharmaceutical innovations may serve as a therapeutic bridge until new chemical entities to replace them are successfully developed. These pharmaceutical maneuvers can be applied to future antibiotics as well. It is clear that a multi-prong therapeutic approach, using a unique combination of pharmacological agents with potentially important antimicrobial and metabolic activities, is an important stratagem against AMR. The challenge ahead for this novel approach consists in identifying the effective concentrations of polyfunctional excipients, as well as “conventional” APIs, that safely achieve pharmaceutical and pharmacological functions.

## Figures and Tables

**Table 1:** Sample composition of an API regimen in the treatment of Gram-negative Sepsis.

	Ingredient	Pharmaceutical Function	Therapeutic Function
1	Ceftolozane	---	β-lactam antibiotic
2	Tazobactam	---	β-lactamase inhibitor
3	krill oil phospholipids	Emulsifier	binds endotoxin; 2nd source of omega-3 fatty acids
4	enriched fish oil triglycerides	drug vehicle	major source of omega-3 fatty acids
5	medium chain triglyceride (MCT) oil	drug vehicle	improves lipid clearance
6	glycerol	tonicity agent	---
7	sodium oleate	co-emulsifier	binds endotoxin?
8	alpha-tocopherol	antioxidant	antioxidant delivery?
9	sterile water for injection	drug vehicle	---

## Abbreviations

AMR	antimicrobial resistance
API	active pharmaceutical ingredient
PK/PD	pharmacokinetic/pharmacodynamics
ICU	intensive care unit
SIRS	systemic inflammatory response syndrome
CRP	C-reactive protein
PGs	prostaglandins
TXAs	thromboxanes
LKT	leukotrienes
FDA	Food and Drug Administration
NSAID	nonsteroidal anti-inflammatory drug
LA	linoleic acid
ARA	arachidonic acid
EPA	eicosapentaenoic acid
DHA	docosahexaenoic acid
KO	krill oil
PL	phospholipids
EP	European Pharmacopoeia
MCT	medium chain triglycerides
LCT	long chain triglycerides
